# Comprehensive Analysis of Competitive Endogenous RNAs Network, Being Associated With Esophageal Squamous Cell Carcinoma and Its Emerging Role in Head and Neck Squamous Cell Carcinoma

**DOI:** 10.3389/fonc.2019.01474

**Published:** 2020-01-21

**Authors:** Donghu Yu, Xiaolan Ruan, Jingyu Huang, Weidong Hu, Chen Chen, Yu Xu, Jinxuan Hou, Sheng Li

**Affiliations:** ^1^Department of Biological Repositories, Zhongnan Hospital of Wuhan University, Wuhan, China; ^2^Human Genetics Resource Preservation Center of Hubei Province, Wuhan, China; ^3^Department of Hematology, Renmin Hospital of Wuhan University, Wuhan, China; ^4^Department of Thoracic Surgery, Zhongnan Hospital of Wuhan University, Wuhan, China; ^5^Department of Radiation and Medical Oncology, Zhongnan Hospital of Wuhan University, Wuhan, China; ^6^Department of Thyroid and Breast Surgery, Zhongnan Hospital of Wuhan University, Wuhan, China

**Keywords:** esophageal squamous cell carcinoma, head and neck squamous cell carcinoma, prognosis, weighted gene co-expression network analysis, competitive endogenous RNAs network

## Abstract

Esophageal squamous cell carcinoma (ESCC) is a common malignancy with poor prognosis and survival rate. To identify meaningful long non-coding RNA (lncRNA), microRNA (miRNA), and messenger RNA (mRNA) modules related to the ESCC prognosis, The Cancer Genome Atlas-ESCC was downloaded and processed, and then, a weighted gene co-expression network analysis was applied to construct lncRNA co-expression networks, miRNA co-expression networks, and mRNA co-expression networks. Twenty-one hub lncRNAs, seven hub miRNAs, and eight hub mRNAs were clarified. Additionally, a competitive endogenous RNAs network was constructed, and the emerging role of the network involved in head and neck squamous cell carcinoma (HNSCC) was also analyzed using several webtools. The expression levels of eight hub genes (TBC1D2, ATP6V0E1, SPI1, RNASE6, C1QB, C1QC, CSF1R, and C1QA) were different between normal esophageal tissues and HNSCC tissues. The expression levels of TBC1D2 and ATP6V0E1 were related to the survival time of HNSCC. The competitive endogenous RNAs network might provide common mechanisms involving in ESCC and HNSCC. More importantly, useful clues were provided for clinical treatments of both diseases based on novel molecular advances.

## Introduction

Esophageal squamous cell carcinoma (ESCC) is the globally predominant pathological type of esophageal cancer (1). For the lack of effective biomarkers, most patients with ESCC are diagnosed at a late stage, which leads to the poor prognosis of ESCC, with a 5-year survival rate of <20% (2, 3). Numerous studies have shown that T stage was the independent factor which influenced the prognosis of ESCC. Besides, most patients with ESCC have a high prevalence of second primary head and neck squamous cell carcinoma (HNSCC) (4). In Taiwan, 15–20% of patients with ESCC may develop a secondary HNSCC (5). Nowadays, it is necessary to do routine screening of head and neck field for the patients with newly diagnosed ESCC and that results in more frequent detection of second primary HNSCC. Therefore, it is of great value to identify the molecular mechanisms related to the development and the prognosis of ESCC, and further research for ESCC-HNSCC pathogenesis is also urgently needed.

Long non-coding RNA (lncRNA) refers to a non-coding RNA transcript with a length >200 nucleotides (6). In recent years, increasing evidences have revealed that multiple lncRNAs can play as potential biomarkers for the prognosis prediction of ESCC, including RNA-PCAT-1 (7), TTN-AS1 (8), and linc00460 (9). However, studies of single lncRNA cannot meet the requirement for exploration of ESCC prognosis. A lncRNA–microRNA (miRNA)–messenger RNA (mRNA) network, which is involved in many important cellular pathways, is badly needed to clarify exact mechanisms.

The competing endogenous RNA (ceRNA) hypothesis was presented by Salmena et al., which stated that mRNAs, lncRNAs, and other non-coding RNAs can act as natural miRNA “sponges” with common MREs to regulate the expression levels of certain genes (10). Nowadays, more and more studies have proven that the ceRNA regulation theory plays an important role in the development of cancer (11). For example, lncRNA-TTN-AS1 was identified to be a target of miR133b, and miR133b can repress the mRNA of fascin homolog 1 in ESCC. Further experiments demonstrated that lncRNA-TTN-AS1 could operate as a ceRNA for binding the microRNA to regulate the expression level of fascin homolog 1 (8).

Although Xue has reported differently expressed lncRNAs, miRNAs, and mRNAs between normal and ESCC tissues (12), the relationships between hub RNAs and important clinical traits had not been rigorously studied. To fulfill these gaps, mRNA co-expression networks, miRNA co-expression networks, and lncRNAs co-expression network were constructed by weighted gene co-expression network analysis (WGCNA) to identify mRNA, miRNA, and lncRNA modules related to T stage in ESCC. WGCNA is a method of mining module information from sequencing data. Under certain conditions, module is defined as a group of genes with similar expression changes in physiological process. This method seems similar to cluster analysis, and the difference is that WGCNA has a biological significance (13). The relationships between the modules and clinical features could be further explored to select candidate biomarkers for cancers. The relationships between lncRNAs and miRNAs, and miRNAs and mRNAs were predicted to build the lncRNA–miRNA–mRNA network, which would provide more information about the mechanisms of ESCC progression, even ESCC-HNSCC pathogenesis.

## Materials and Methods

### Data Collection and Processing

**Figure 1:**
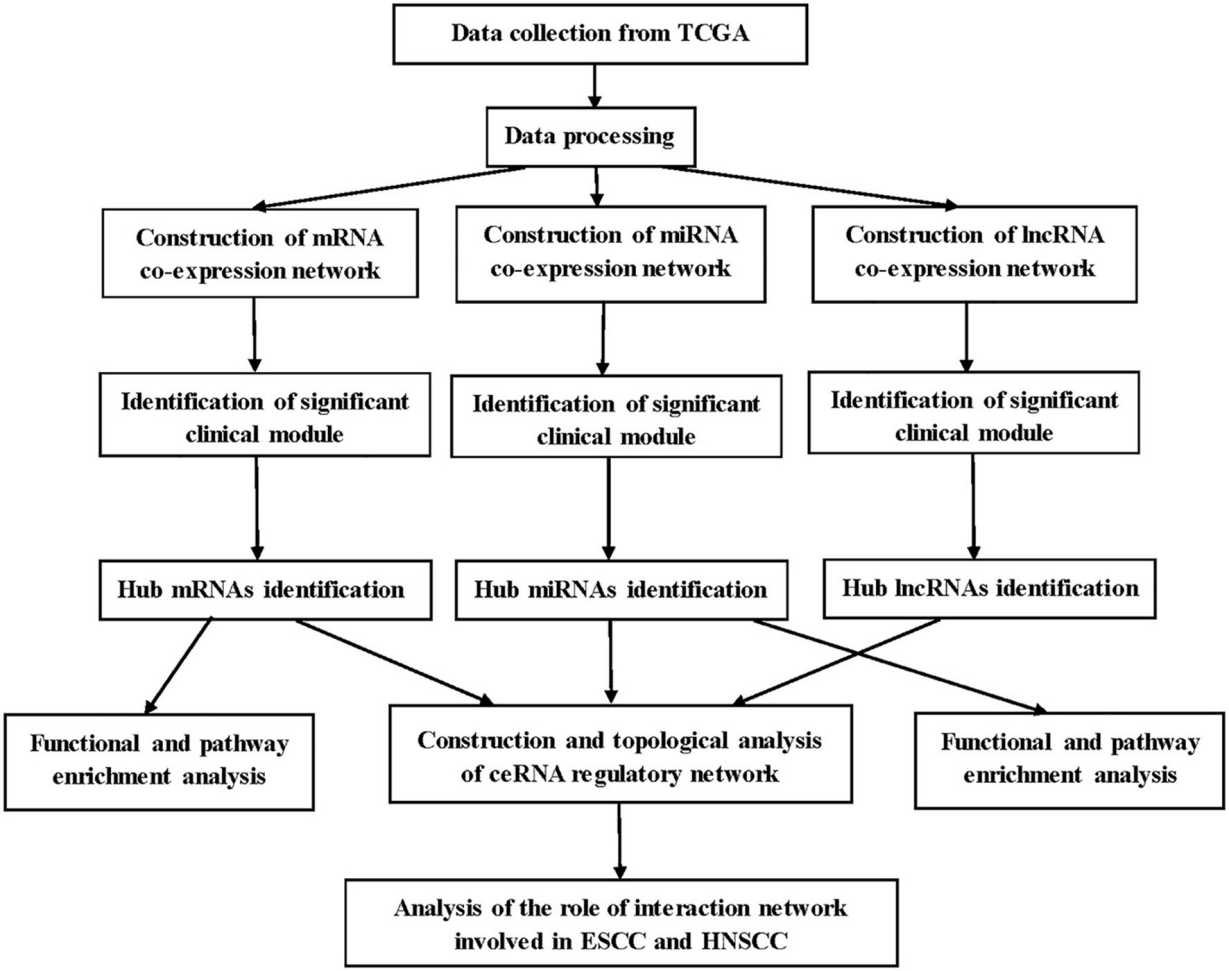
Flow chart of data preparation, processing, and analysis.

A brief workflow for this study is shown in Figure 1. The RNA sequencing data of 95 samples with ESCC were retrieved from The Cancer Genome Atlas (TCGA) data portal (https://cancergenome.nih.gov/), which had been derived from the IlluminaHiSeq_RNASeq and the IlluminaHiSeq_miRNASeq sequencing platforms. Ninety-five samples were divided into two groups: 17 normal samples and 78 tumor samples. Gene expression profiles (GSE20437 and GSE38129) related to ESCC, which were downloaded for the validation from Gene Expression Omnibus database (https://www.ncbi.nlm.nih.gov/geo/), provided validation for selected hub mRNAs. The details of GSE20437 and GSE38129 are listed in Table S1. All datasets were normalized with quantile normalization. Analysis of variance were performed for TCGA-ESCC-mRNA and TCGA-ESCC-lncRNA. We chose the top 25% most variant mRNAs (4,938 mRNAs) and the top 25% most variant lncRNAs (3,712 genes) for constructing networks, while we did not do pretreatment for miRNA expression profile due to the small number of miRNAs (1,881 miRNAs).

### Construction of Co-expression Networks

WGCNA was used to construct mRNA, miRNA, and lncRNA co-expression networks (14). The processes for constructing co-expression networks were similar. Thus, we took the construction of weighted mRNA co-expression networks as an example. First, a matrix of similarity was constructed by calculating the correlations of the processed genes. Then, an appropriate power of β was chosen as the soft-thresholding parameter to construct a scale-free network. Next, the adjacency was transformed into a topological overlap matrix (TOM) using TOM similarity, and the corresponding dissimilarity (1—TOM) was figured and the dissimilarity of module eigengenes (MEs) estimated. Last, the mRNAs with similar expression levels were categorized into the same module by DynamicTreeCut algorithm (15).

### Identification of Clinically Significant Modules

The clinical trait we were concerned was T stage in ESCC patients and key modules which needed to be found in three networks separately. Above all, we worked out the relationship between clinical phenotype and MEs. MEs were deemed to represent the expression levels of all mRNAs, miRNAs, or lncRNAs in the related module. In addition, mediated *P*-value of each mRNA, miRNA, or lncRNA was calculated, and then, we worked out gene, miRNA, or lncRNA significance (GS = lg *P*). Finally, we selected the most clinically significant module according to module significance, which was the average GS of mRNAs, miRNAs, or lncRNAs involved in the related module. Besides, the connectivity of module was measured by absolute value of the Pearson's correlation, and the relationships between clinical trait and mRNAs, miRNAs, or lncRNAs were measured by absolute value of the Pearson's correlation. To build a ceRNA regulatory network in ESCC better, two modules in each co-expression network were selected. The RNA expression levels in one module were positively correlated with the clinical trait (T stage), and the RNA expression levels in the other module were negatively correlated with the T stage of ESCC.

### Functional and Pathway Enrichment Analysis

The Database for Annotation, Visualization, and Integrate Discovery (DAVID) (https://david.ncifcrf.gov/) is a database for several kinds of functional annotation (16). With the help of Database for Annotation, Visualization, and Integrate Discovery, we identified biological meaning of the mRNAs in hub modules according to false discovery rate (FDR) <0.05. Gene Ontology (GO) includes three terms: biological process (BP), cellular component (CC), and molecular function (MF); GO (BP, CC, MF) and Kyoto Encyclopedia of Genes and Genomes (KEGG) enrichment analyses for the miRNAs in the hub modules were conducted using mirPath v.3, an online tool for miRNA pathway analysis (17). GO (BP, CC, MF) and KEGG enrichment analyses for the lncRNAs in the hub modules were conducted using co-lncRNA, a web-based computational tool that allows users to identify GO annotations and KEGG pathways that may be affected by co-expressed protein-coding genes of a single or multiple lncRNAs (18).

### Identification and Validation of Hub mRNAs in ESCC

To identify real hub mRNAs associated with the development of ESCC, three methods were used to screen candidate mRNAs. First, the mRNAs that have high connectivity with module and selected phenotype were chosen as candidate genes in hub module [|cor. module membership| (|MM|) > 0.35]. Then, the protein/gene interactions for the mRNAs in each hub module were analyzed using STRING (19), and the mRNAs connected with more than four nodes in PPI network were selected as candidate mRNAs for further study. Next, survival analysis was performed for the mRNAs in each hub module by survival package in R, and the mRNAs with *P* < 0.05 were considered to be associated with overall survival in ESCC. Then, the common candidate mRNAs in three parts were considered as hub mRNAs. To verify our results, GSE20347 (including 17 normal esophageal tissues and 17 ESCC tissues) and GSE38129 (including 30 normal esophageal tissues and 30 ESCC tissues) were used to validate the different expression levels of hub mRNAs between normal tissues and ESCC tissues. Under the threshold of |log_2_ FC| > 1.5 and FDR < 0.05, differently expressed genes (DEGs) were selected by “limma” package in R in two datasets, separately. OSescc, containing survival data from GSE53625 and TCGA and giving users the ability to create publication-quality Kaplan–Meier plots (20), was used to further explore the prognostic biomarker in the dataset GSE53625 (21).

### Identification Hub miRNAs and lncRNAs

The interactions between lncRNA and miRNA, and mRNA and miRNA could be predicted. As for selecting hub miRNAs, TargetScan (http://www.targetscan.org/) was employed to predict candidate miRNAs for hub mRNAs (22, 23), and context++ score of TargetScan > 0.4 were selected as threshold. Then, the common candidate miRNAs with |MM| > 0.4 in hub modules and prediction by TargetScan was defined as real hub miRNAs. LncBase (http://carolina.imis.athena-innovation.gr/diana_tools/web/index.php?r=lncbasev2) was used to predict lncRNA and miRNA interactions (24), and the score of LncBase > 0.7 was selected as threshold. The common candidate lncRNAs with |MM| > 0.7 in hub modules and prediction by LncBase were defined as real hub lncRNAs.

### Construction and Topological Analysis of ceRNA Regulatory Network in ESCC

According to the prediction of TargetScan and LncBase, the interactions were used to construct the lncRNA–miRNA–mRNA network applying the Cytoscape software, and the interaction between genes was also demonstrated from STRING (25). It is well-known that hub nodes play critical roles in biological networks. Simultaneously, all node degrees of the lncRNA–miRNA–mRNA network were calculated by “NetworkAnalyzer” in Cytoscape.

### The Prognostic Factors of ceRNA Network in ESCC and HNSCC

Survival analysis was performed for the mRNAs/miRNAs/lncRNAs in ceRNA network by survival package in R, and the threshold was selected as *P* < 0.05. In addition, to explore the role of the interaction network in HNSCC, UALCAN (http://ualcan.path.uab.edu/) was used to find the different expression levels of hub genes between normal tissues and cancer tissues. UALCAN is a useful online tool for analyzing cancer transcriptome data, which is based on public cancer transcriptome data (TCGA and MET500 transcriptome sequencing) (26). OncomiR (http://www.oncomir.org/), an online resource for exploring miRNA dysregulation in cancer based on TCGA, was used to find the different expression levels of hub miRNAs between normal tissues and cancer tissues (27). To explore the expression levels of hub lncRNAs in normal and HNSCC samples, independent *t*-test was performed for the hub lncRNAs with the dataset of TCGA-HNSCC-lncRNA. Besides, OncoLnc (http://www.oncolnc.org/), containing survival data from 21 cancer studies performed by TCGA and giving users the ability to create publication-quality Kaplan–Meier plots, was used to explore the relationship between the expression levels of hub mRNAs/miRNAs/lncRNAs and the survival time of HNSCC (28).

### Functional Annotation of the Hub Genes

Gene Set Enrichment Analysis (GSEA) was performed for hub mRNAs in TCGA-ESCC (29). In TCGA-ESCC, according to the median expression of this hub gene, 119 cases were classified into high- and low-expression group (high group, *n* = 60; low group, *n* = 59). Gene size > 100, |ES| > 0.6, nominal *P* < 0.05, and FDR < 25% were chosen as the cutoff criteria. Besides, Spearman correlation analysis was performed to explore pairwise gene expression correlation for hub genes in TCGA-ESCC. We calculated correlation coefficient absolute values, and the top 300 hub genes were selected for functional enrichment analysis. Based on the results, the potential functions of each hub gene were predicted, and the method thus bore the name of “guilt of association” (30).

## Results

### Weighted Co-expression Networks Construction and Key Modules Identification

**Figure 2:**
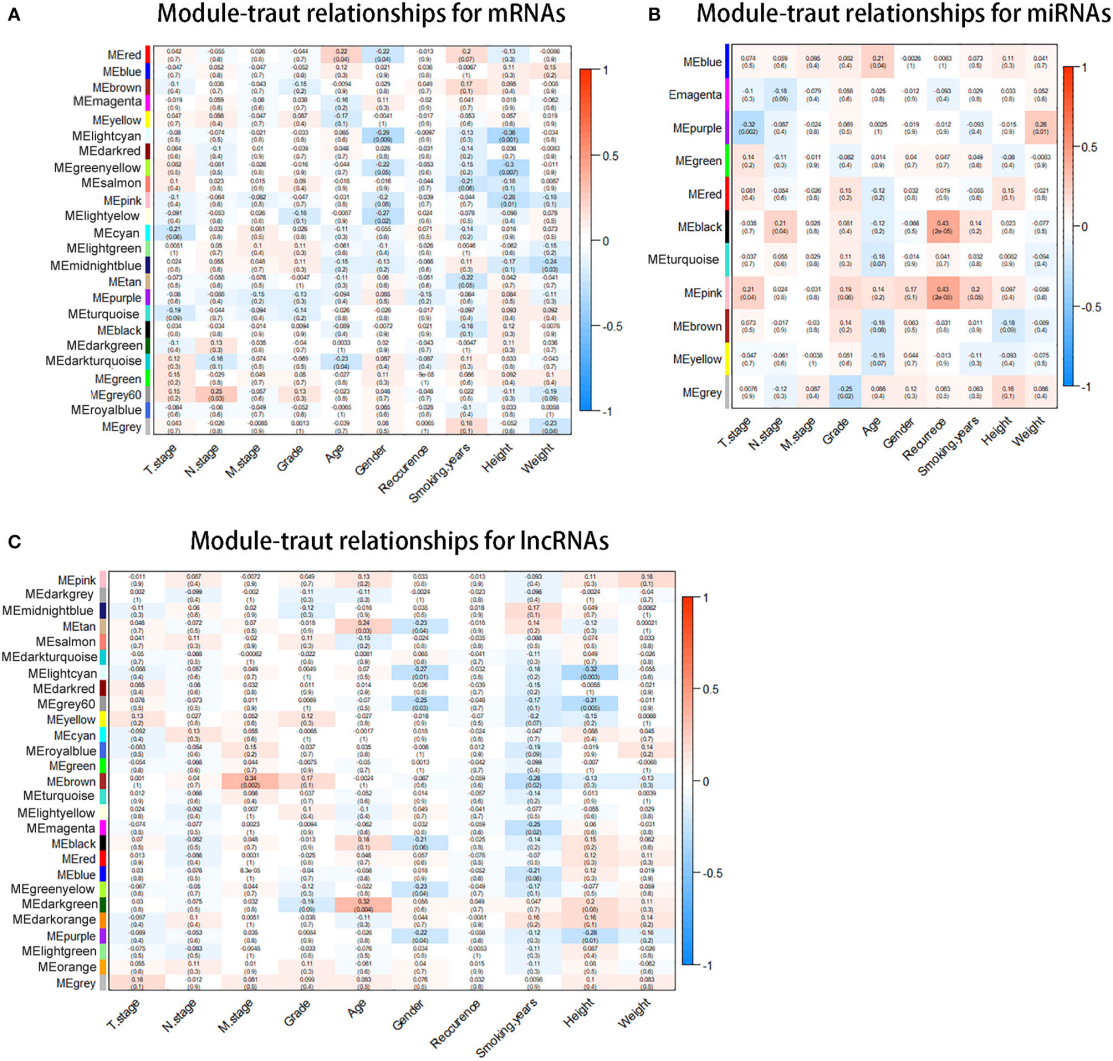
Identification of modules associated with the clinical traits of esophageal squamous cell carcinoma (ESCC). **(A)** Distribution of average messenger RNA (mRNA) significance and errors in the modules associated with the T stage in ESCC. **(B)** Distribution of average microRNA (miRNA) significance and errors in the modules associated with the T stage. **(C)** Distribution of average long non-coding RNA (lncRNA) significance and errors in the modules associated with the T stage of esophageal squamous cell carcinoma (ESCC).

With the method of average linkage hierarchical clustering, the samples of TCGA-ESCC were well clustered. To ensure a scale-free network, power of β = 5 (scale-free *R*^2^ = 0.949) was selected as the soft-thresholding parameter for mRNA co-expression networks (Figure S1A). Power of β = 3 (scale-free *R*^2^ = 0.939) was selected for miRNA co-expression networks (Figure S1B). Power of β = 5 (scale-free *R*^2^ = 0.935) was selected for lncRNA co-expression networks (Figure S1C). The clustering dendrograms of the mRNAs (Figure S2A), miRNAs (Figure S2B), and lncRNAs (Figure S2C) were generated. By “WGCNA” package in R, the mRNAs, the miRNAs, and the lncRNAs, which had similar expression levels, were divided into modules to construct co-expression networks, separately. In mRNA co-expression networks, green module (GS = 0.15; containing 279 mRNAs) and cyan module (GS = −0.21; containing 92 mRNAs) showed the highest correlation with T stage of ESCC (Figure 2A). In miRNA co-expression networks, pink module (GS = 0.21; containing 46 miRNAs) and purple module (GS = −0.32; containing 38 miRNAs) showed the highest correlation with T stage of ESCC (Figure 2B). In lncRNA co-expression networks, yellow module (GS = 0.13; containing 180 lncRNAs) and midnight blue module (GS = −0.11; containing 71 lncRNAs) showed the highest correlation with T stage of ESCC (Figure 2C). Six modules from three networks were picked for following analysis as the clinically significant modules.

### Functional and Pathway Enrichment Analysis

**Figure 3:**
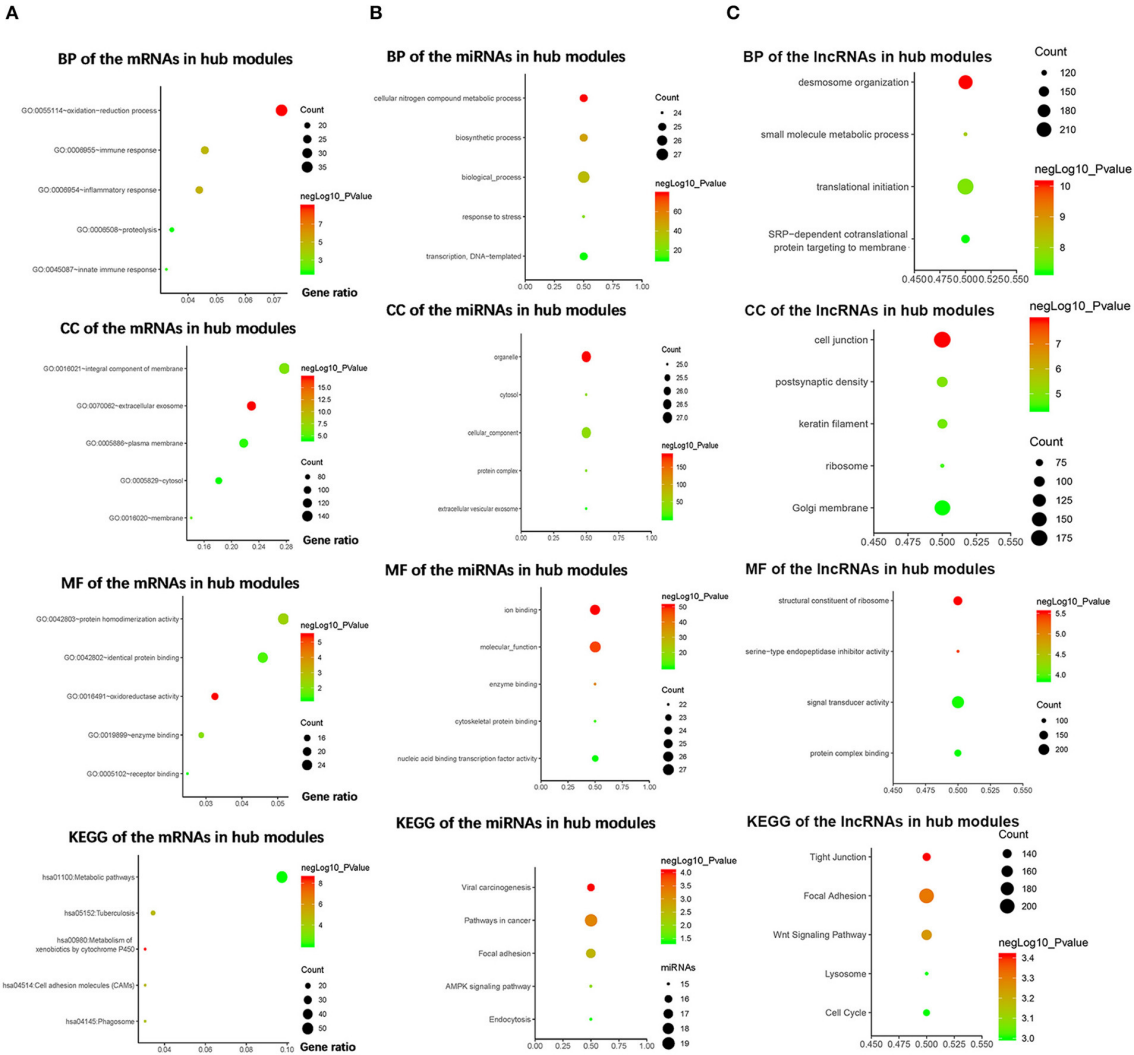
Bioinformatics analysis of the messenger RNAs (mRNAs) and the microRNAs (miRNAs) in hub modules. **(A)** Gene Ontology (GO) analysis and Kyoto Encyclopedia of Genes and Genomes (KEGG) pathway enrichment of the mRNAs in green and cyan modules. **(B)** GO analysis and KEGG pathway enrichment of the miRNAs in pink and purple modules. **(C)** GO analysis and KEGG pathway enrichment of the lncRNAs in the yellow and midnight blue modules.

To explore the biological functions of the mRNAs in hub modules, the mRNAs were categorized into BP, CC, and MF. The outcome of GO and KEGG enrichment of the mRNAs in green and cyan module is shown in Figure 3A. The mRNAs in BP were generally enriched in oxidation–reduction process, immune response, inflammatory response, proteolysis, and innate immune response; the mRNAs in CC were mainly focused on integral component of membrane, extracellular exosome, plasma membrane, cytosol, and membrane; the mRNAs in MF were significantly focused on protein homodimerization activity, identical protein binding, oxidoreductase activity, enzyme binding, and receptor binding. The top five significantly enriched pathways in green and cyan module were metabolic pathways, tuberculosis, metabolism of xenobiotics by cytochrome P450, cell adhesion molecules, and phagosome. Top enriched GO terms for the miRNAs in pink and purple modules were the following: biological process, cellular nitrogen compound metabolic process, biosynthetic process, transcription, DNA-templated and response to stress in BP; organelle, cellular component, cytosol, protein complex, and extracellular vesicular exosome in CC; and molecular function, ion binding, nucleic acid binding transcription factor activity, enzyme binding, and cytoskeletal protein binding in MF. The pathway analysis was also performed for the miRNAs in hub modules. The top five significantly enriched pathways were pathways in cancer, focal adhesion, viral carcinogenesis, AMPK signaling pathway, and endocytosis (Figure 3B). Top enriched GO terms for the lncRNAs in yellow and midnight blue modules were as follows: desmosome organization, small molecule metabolic process, translational initiation, signal-recognition particle-dependent co-translational protein targeting to membrane, and keratinocyte differentiation in BP; Golgi membrane, cell junction, postsynaptic density, keratin filament, and ribosome in CC; signal transducer activity, structural constituent of ribosome, protein complex binding, serine-type endopeptidase inhibitor activity, and metallopeptidase activity in MF. The pathway analysis was also performed for the lncRNAs in hub modules. The top five significantly enriched pathways were focal adhesion, Wnt signaling pathway, tight junction, cell cycle, and lysosome (Figure 3C).

### Identification and Validation of Hub mRNAs in ESCC

**Figure 4:**
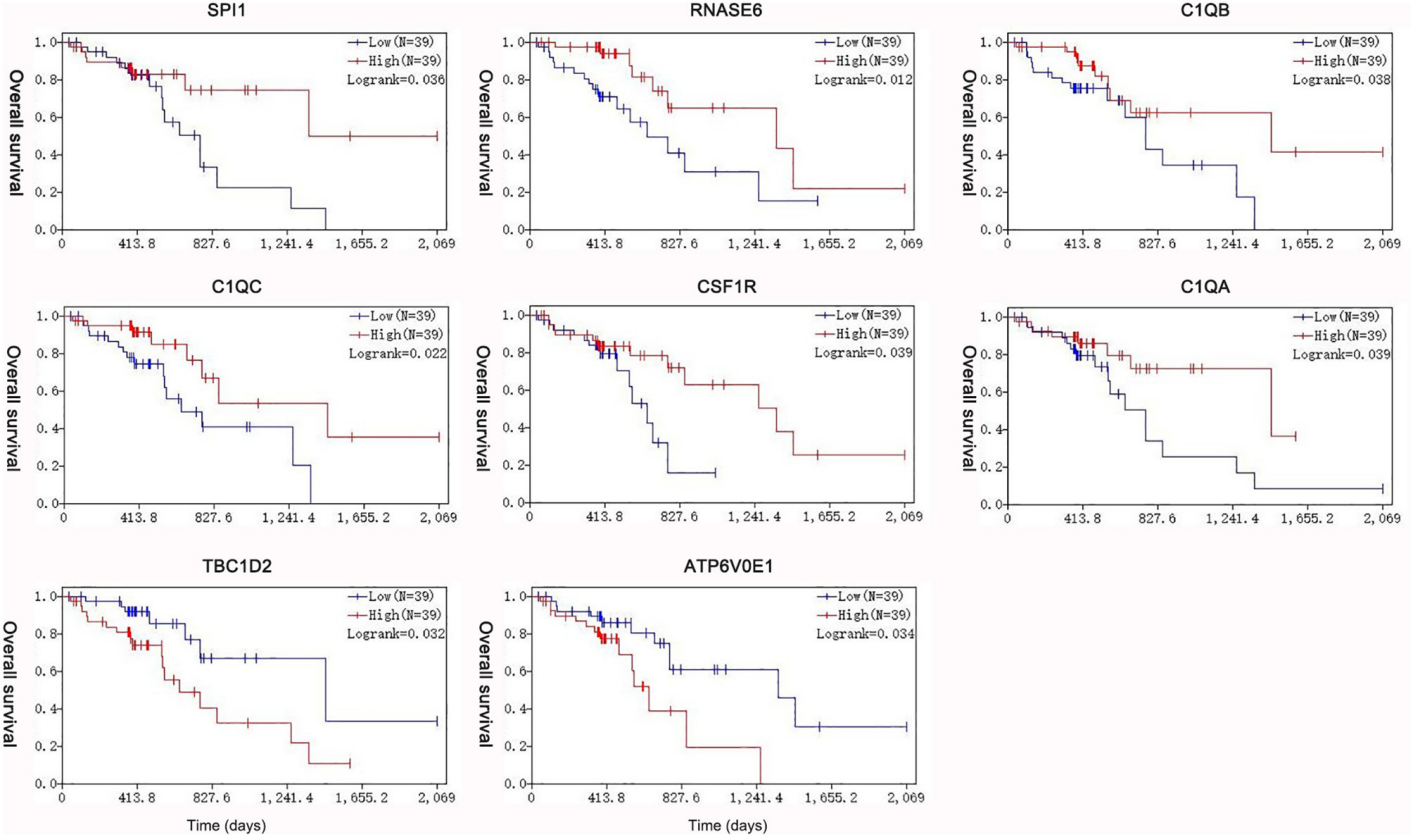
Survival analysis of the association between the expression levels of hub mRNAs based on The Cancer Genome Atlas-esophageal squamous cell carcinoma (TCGA-ESCC). The expression levels of SPI1, RNASE6, C1QB, C1QC, CSF1R, and C1QA were positively correlated with the overall survival. The expression levels of TBC1D2 and ATP6V0E1 were negatively correlated with the overall survival of ESCC.

**Figure 5:**
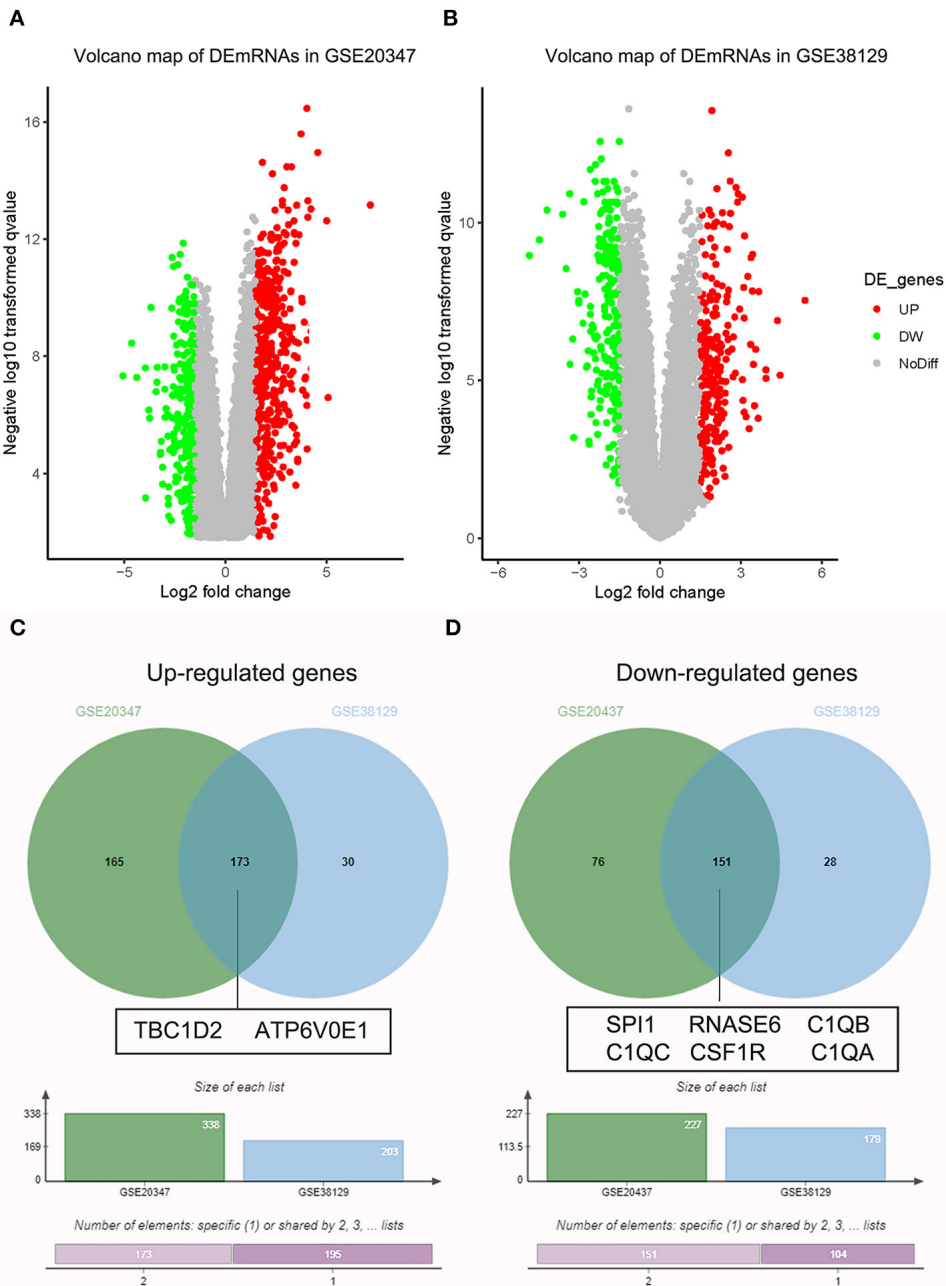
Validation of hub messenger RNAs (mRNAs) in esophageal squamous cell carcinoma (ESCC). **(A)** Volcano plot visualizing differently expressed genes (DEGs) in GSE20347 (17 normal samples and 30 ESCC samples). **(B)** Volcano plot visualizing DEGs in GSE38129 (30 normal samples and 30 ESCC samples). **(C)** Identification of common upregulated genes between DEGs of GSE20347 and GSE38129. **(D)** Identification of common downregulated genes between DEGs of GSE20347 and GSE38129 by overlapping them.

Under the threshold of |MM| > 0.35, 103 mRNAs in cyan module and 17 mRNAs in green module were considered as candidate genes. Then, the relationship between mRNAs in each module was identified from STRING (Figure S3), and we calculated the connectivity degree of each node in PPI. Sixty mRNAs in green module and 148 mRNAs with degrees ≥4 were considered as candidate mRNAs because they interacted with more proteins. As for the survival analysis, 17 mRNAs in green module and 29 mRNAs in cyan module were identified to be related to the overall survival in ESCC. To identify the common mRNAs in three parts, we performed Venn diagram by online tool jvenn (http://jvenn.toulouse.inra.fr/app/example.html) (Figure S4). Two mRNA (TBC1D2 and ATP6V0E1) in green module and six mRNAs (SPI1, RNASE6, C1QB, C1QC, CSF1R, and C1QA) in cyan module were considered as real hub mRNAs, and they were closely related to the overall survival in ESCC (Figure 4). The corresponding MM and GS of the hub mRNAs in hub modules are shown in Table 1. GSE20347 and GSE38129 were used to validate the different expression levels of hub mRNAs between normal tissues and ESCC tissues with “limma” package in R. The results showed that TBC1D2 and ATP6V0E1 were significantly downregulated in ESCC (log_2_ FC > 1.5 and FDR < 0.05), while SPI1, RNASE6, C1QB, C1QC, CSF1R, and C1QA are significantly downregulated (log_2_ FC < −1.5 and FDR < 0.05) (Figure 5). It is a pity that no other significant difference was observed in the prognostic analysis for the biomarkers in GSE53625 except for TBC1D2 (log-rank *P* = 0.028615) from OSescc.

**Table 1:** The corresponding GS and MM of the hub mRNAs, hub miRNAs and lncRNAs in hub modules.

	**ID**	**Module**	**GS**	**MM**
mRNA	TBC1D2	Green	0.066802	0.396126
mRNA	ATP6V0E1	Green	0.068227	0.443309
mRNA	SPI1	Cyan	−0.26978	0.92313
mRNA	RNASE6	Cyan	−0.14359	0.902145
mRNA	C1QB	Cyan	−0.13492	0.84643
mRNA	C1QC	Cyan	−0.12612	0.835847
mRNA	CSF1R	Cyan	−0.16647	0.830564
mRNA	C1QA	Cyan	−0.15212	0.826306
miRNA	hsa-miR-515-5p	Pink	0.175606	0.747637
miRNA	hsa-miR-519e-5p	Pink	0.15501	0.49922
miRNA	hsa-miR-6769b-5p	Pink	0.147596	0.410585
miRNA	hsa-miR-519d-5p	Pink	0.013167	0.541514
miRNA	hsa-miR-4707-3p	Purple	−0.24545	0.7482
miRNA	hsa-miR-6756-5p	Purple	−0.28336	0.908069
miRNA	hsa-miR-650	Purple	−0.32189	0.94761
lncRNA	RP5-1029K10.2	Yellow	0.173204	0.816681
lncRNA	ETV5-AS1	Yellow	0.13791	0.78535
lncRNA	RP11-440L14.1	Yellow	0.131855	0.84422
lncRNA	RP5-1184F4.5	Yellow	0.131485	0.854835
lncRNA	AC226118.1	Yellow	0.111381	0.76165
lncRNA	RP3-470B24.5	Yellow	0.108569	0.870981
lncRNA	RP5-1125A11.7	Yellow	0.107931	0.741911
lncRNA	CTD-2023N9.1	Yellow	0.104444	0.900808
lncRNA	RP11-332H14.2	Yellow	0.09411	0.797046
lncRNA	XIST	Yellow	0.089522	0.401167
lncRNA	AC141928.1	Yellow	0.072768	0.73042
lncRNA	RP5-1054A22.4	Yellow	0.072657	0.868935
lncRNA	C1orf213	Yellow	0.066348	0.746537
lncRNA	PSMG3-AS1	Yellow	0.063239	0.798
lncRNA	AC016735.1	Yellow	0.056561	0.704278
lncRNA	RP11-2H3.6	Yellow	0.035953	0.826073
lncRNA	RP11-504P24.8	Yellow	0.023092	0.706706
lncRNA	CTD-3018O17.3	Yellow	0.009725	0.726373
lncRNA	LINC01355	Yellow	0.001995	0.730689
lncRNA	RP11-327F22.6	Midnight blue	−0.04346	0.733582
lncRNA	RP11-275I4.2	Midnight blue	−0.06852	0.711478

*miRNA, microRNA; lncRNA, long non-coding RNA; GS, gene significance; MM, module membership*.

### Identification of Hub miRNAs and lncRNAs

Based on the MM of miRNA co-expression network and the prediction by TargetScan (Table 2), seven miRNAs (hsa-miR-519e-5p, hsa-miR-519d-5p, hsa-miR-515-5p, hsa-miR-6756-5p, hsa-miR-6769b-5p, hsa-miR-4707-3p, and hsa-miR-650) were defined as real hub miRNAs. Based on the MM of lncRNA co-expression network and the prediction by LncBase (Table 3), 21 lncRNAs (RP11-275I4.2, RP11-327F22.6, LINC01355, CTD-3018O17.3, RP11-504P24.8, RP11-2H3.6, AC016735.1, PSMG3-AS1, C1orf213, RP5-1054A22.4, AC141928.1, XIST, RP11-332H14.2, CTD-2023N9.1, RP5-1125A11.7, RP3-470B24.5, AC226118.1, RP5-1184F4.5, RP11-440L14.1, ETV5-AS1, and RP5-1029K10.2) were considered as hub lncRNAs. The corresponding MM and GS of the hub miRNAs and the hub lncRNAs in hub modules are shown in Table 1.

**Table 2:** The prediction of the interaction of hub mRNAs and hub miRNAs by Targetscan.

**miRNA**	**Target gene**	**Context++ score of TargetScan**
hsa-miR-519e-5p	RNASE6	−0.48
hsa-miR-515-5p	RNASE6	−0.48
hsa-miR-519d-5p	RNASE6	−0.45
hsa-miR-6756-5p	C1QA	−0.64
hsa-miR-6769b-5p	C1QA	−0.4
hsa-miR-4707-3p	TBC1D2	−0.59
hsa-miR-519d-5p	ATP6V0E1	−0.4
hsa-miR-650	ATP6V0E1	−0.59

*mRNA, messenger RNA; miRNA, microRNA*.

**Table 3:** The prediction of the interaction of hub lncRNAs and hub miRNAs by LncBase.

**lncRNA**	**Target miRNA**	**The score of LncBase**
XIST	hsa-miR-519e-5p	0.951
CTD-2023N9.1	hsa-miR-519e-5p	0.711
RP5-1184F4.5	hsa-miR-519e-5p	0.71
RP11-440L14.1	hsa-miR-519e-5p	0.987
RP11-332H14.2	hsa-miR-519e-5p	0.774
ETV5-AS1	hsa-miR-519e-5p	0.803
RP11-327F22.6	hsa-miR-519e-5p	0.706
AC141928.1	hsa-miR-519e-5p	0.707
AC016735.1	hsa-miR-519e-5p	0.779
RP5-1054A22.4	hsa-miR-519d-5p	0.726
RP11-327F22.6	hsa-miR-519d-5p	0.712
XIST	hsa-miR-515-5p	0.949
CTD-2023N9.1	hsa-miR-515-5p	0.711
RP5-1184F4.5	hsa-miR-515-5p	0.736
RP11-440L14.1	hsa-miR-515-5p	0.989
RP11-332H14.2	hsa-miR-515-5p	0.782
ETV5-AS1	hsa-miR-515-5p	0.767
AC141928.1	hsa-miR-515-5p	0.715
AC016735.1	hsa-miR-515-5p	0.797
XIST	hsa-miR-6756-5p	0.948
RP5-1029K10.2	hsa-miR-6756-5p	0.944
PSMG3-AS1	hsa-miR-6756-5p	0.711
AC226118.1	hsa-miR-6756-5p	0.7
C1orf213	hsa-miR-6756-5p	0.919
RP5-1125A11.7	hsa-miR-6756-5p	0.73
LINC01355	hsa-miR-6756-5p	0.773
RP11-2H3.6	hsa-miR-6769b-5p	0.919
AC226118.1	hsa-miR-6769b-5p	0.703
CTD-3018O17.3	hsa-miR-6769b-5p	0.848
RP11-275I4.2	hsa-miR-6769b-5p	0.991
RP11-440L14.1	hsa-miR-4707-3p	0.822
RP3-470B24.5	hsa-miR-650	0.815
C1orf213	hsa-miR-650	0.716
CTD-3018O17.3	hsa-miR-650	0.762
RP11-504P24.8	hsa-miR-650	0.974

*lncRNA, long non-coding RNA; miRNA, microRNA*.

### Construction and Topological Analysis of ceRNA Regulatory Network in ESCC

**Figure 6:**
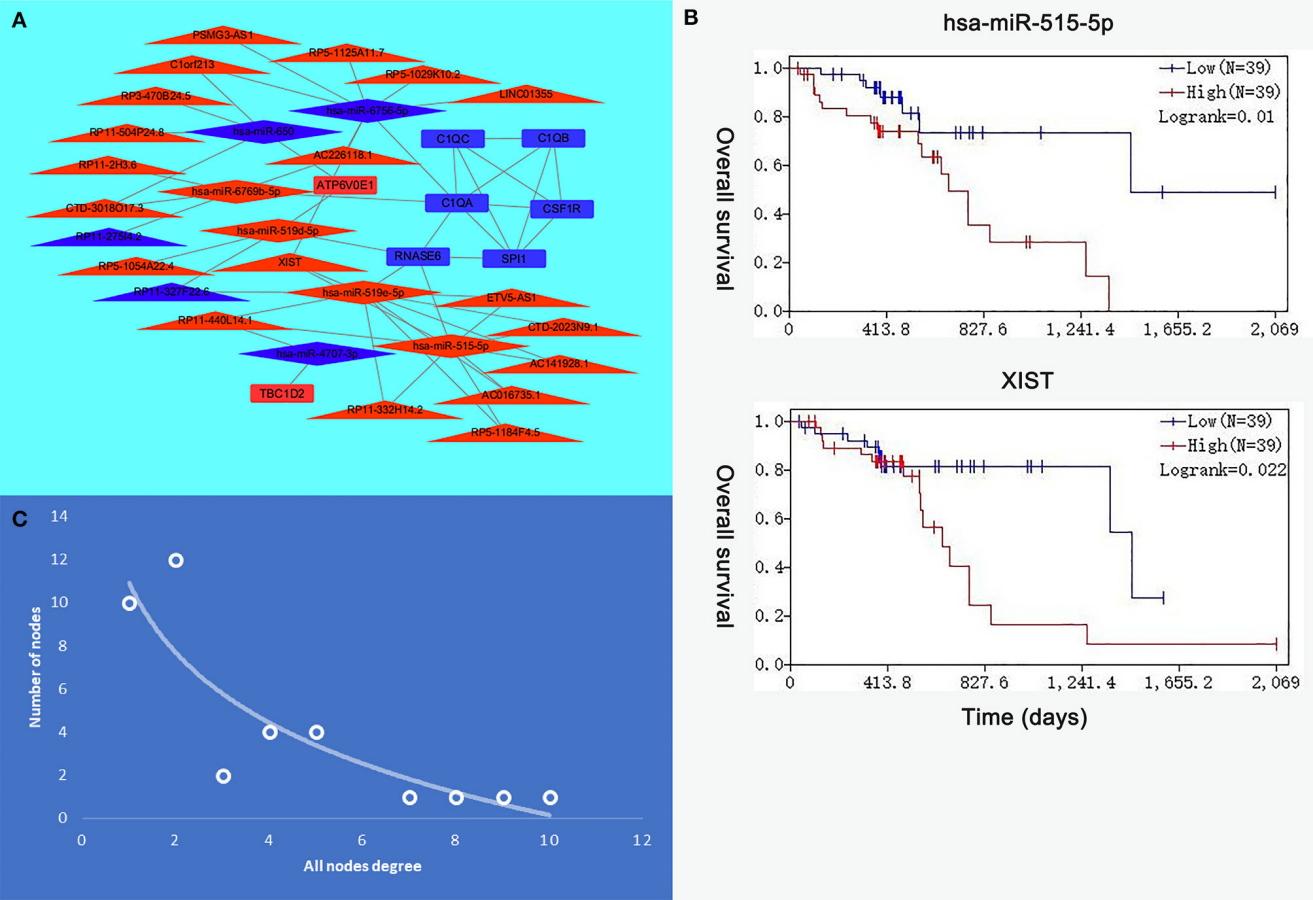
The interaction network of hub microRNAs (miRNAs) and hub genes. **(A)** The view of the long non-coding RNA (lncRNA)–miRNA–messenger RNA (mRNA) network. The triangle represents lncRNAs, the rhombus represents miRNAs, and the rectangle represents mRNAs. **(B)** The expression levels of hsa-miR-515-5p and XIST were negatively correlated with the overall survival. **(C)** All node degree analysis reveals specific properties of the lncRNA–miRNA–mRNA network.

Eight genes (SPI1, RNASE6, C1QB, C1QC, CSF1R, C1QA, TBC1D2, and ATP6V0E1), seven miRNAs (hsa-miR-519e-5p, hsa-miR-519d-5p, hsa-miR-515-5p, hsa-miR-6756-5p, hsa-miR-6769b-5p, hsa-miR-4707-3p, and hsa-miR-650), and 21 lncRNAs (RP11-275I4.2, RP11-327F22.6, LINC01355, CTD-3018O17.3, RP11-504P24.8, RP11-2H3.6, AC016735.1, PSMG3-AS1, C1orf213, RP5-1054A22.4, AC141928.1, XIST, RP11-332H14.2, CTD-2023N9.1, RP5-1125A11.7, RP3-470B24.5, AC226118.1, RP5-1184F4.5, RP11-440L14.1, ETV5-AS1, and RP5-1029K10.2) were involved in this interaction network. The lncRNA–miRNA–mRNA network is shown in Figure 6A. Besides, all node degrees of the network were calculated (Table S2 and Figure 6C). According to the previous studies, a node with degree exceeding 5 was defined as a hub node (31, 32). In our study, eight nodes (including three mRNAs and five miRNAs) were selected as hub nodes. In addition, we calculated the number of the relationship pairs of miRNA–mRNA and lncRNA–miRNA, and the results are shown in Table 4. We found that three miRNAs (hsa-miR-519e-5p, hsa-miR-515-5p, and hsa-miR-6756-5p) not only had higher node degrees but also had a higher number of miRNA–mRNA and lncRNA–miRNA pairs. The results suggested that the miRNAs (hsa-miR-519e-5p, hsa-miR-515-5p, and hsa-miR-6756-5p) might play essential roles in ESCC progression, which would be considered as the key miRNAs.

**Table 4:** The number of lncRNA–miRNA and miRNA–mRNA pairs.

**Number**	**Name**	**lncRNA–miRNA pairs**	**miRNA–mRNA pairs**	**Total number**
1	hsa-miR-519e-5p	9	1	10
2	hsa-miR-515-5p	8	1	9
3	hsa-miR-6756-5p	7	1	8

*lncRNA, long non-coding RNA; miRNA, microRNA*.

### The Prognostic Factors of ceRNA Network in ESCC and HNSCC

**Figure 7:**
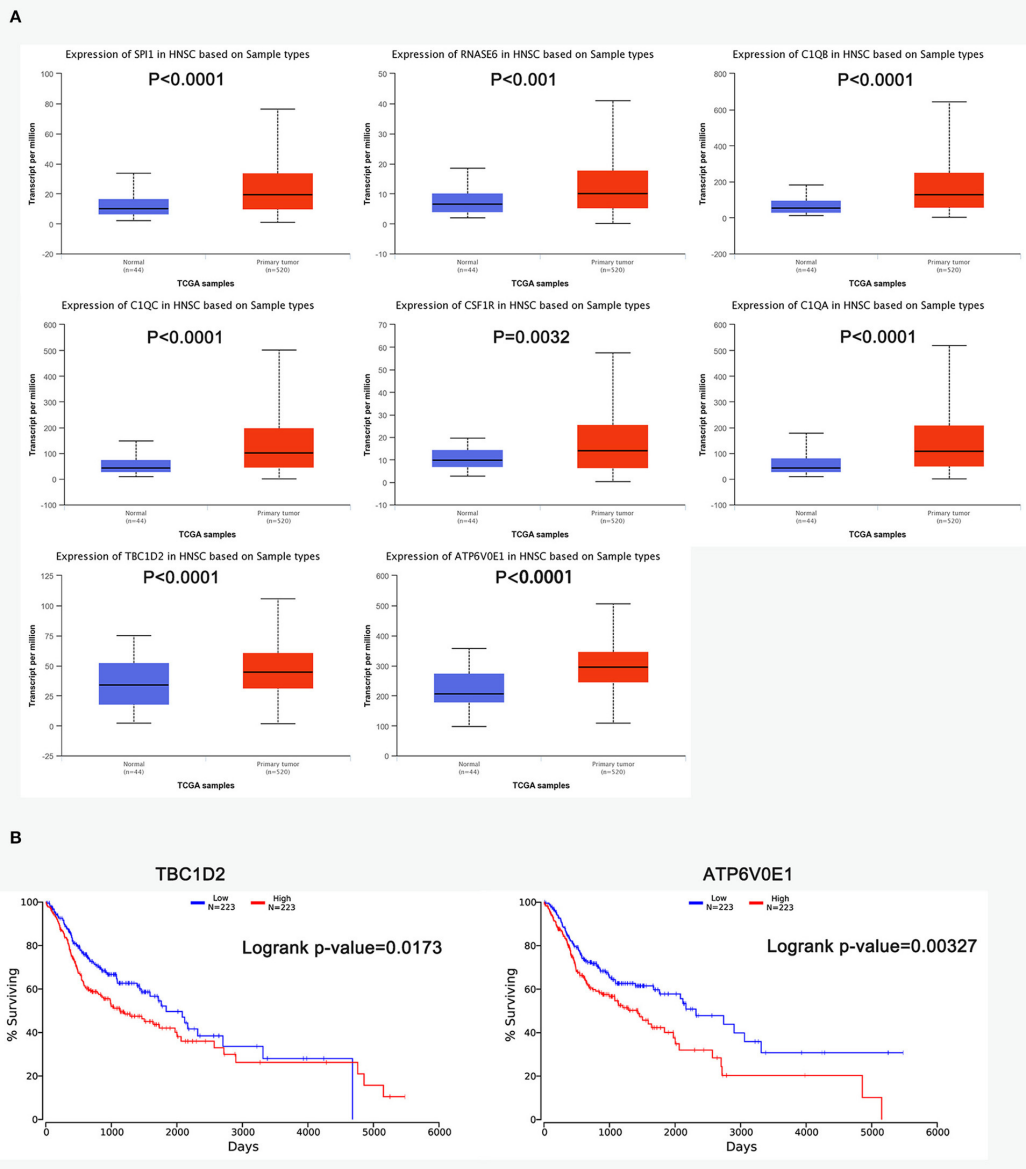
The prognostic factors of competing endogenous RNA (ceRNA) network in head and neck squamous cell carcinoma (HNSCC). **(A)** Gene expression levels between normal and tumor samples [based on The Cancer Genome Atlas (TCGA)-HNSCC data in UALCAN]. **(B)** TBC1D2 and ATP6V0E1 were identified to be related to the overall survival of HNSCC from OncoLnc.

The R survival package was used for survival analysis for all RNAs in the ceRNA network. Because the overall survival of mRNAs was performed to select hub mRNAs (*P* < 0.05), the mRNAs in the ceRNA network were significantly associated with overall survival of ESCC. Through the Kaplan–Meier curve analysis for TCGA-ESCC, one miRNA (hsa-miR-515-5p) and one lncRNA (XIST) were found to be significantly associated with overall survival. We found that the expression levels of the hsa-miR-515-5p miRNA and XIST lncRNA were negatively correlated with the overall survival rate (*P* < 0.05; Figure 6B). Besides, some databases were used to explore the role of the interaction network in HNSCC. The levels of eight genes (SPI1, RNASE6, C1QB, C1QC, CSF1R, C1QA, TBC1D2, and ATP6V0E1) expression were higher in tumor samples from UALCAN (Figure 7A). The results showed that the expression levels of the hub miRNAs/lncRNAs between normal and HNSCC tissues had no obvious difference. For the relationship between hub mRNAs/miRNAs/lncRNAs expression levels and the prognosis of HNSCC from OncoLnc, TBC1D2 and ATP6V0E1 negatively correlated with overall survival of HNSCC (Figure 7B). It is a pity that no other significant difference was observed in the prognostic analysis for the hub miRNAs/lncRNAs in HNSCC.

### Functional Annotation of the Hub Genes

GSEA was performed to identify the lurking mechanisms related to ESCC progression of eight hub genes. As shown in Table S3, ESCC samples in TBC1D2 high-expression group were most significantly enriched in translational initiation molecules; ESCC samples in ATP6V0E1, SPI1, RNASE6, C1QB, C1QC, CSF1R, and C1QA high-expression groups were most significantly enriched in adaptive immune response (Tables S4–S10). Based on the analysis of guilt of association, we identified that the hub genes were essential for T-cell activation, and they mainly played important roles in leukocyte cell–cell adhesion, regulation of lymphocyte activation, and T-cell receptor complex (Figure S5).

## Discussion

Although some certain chemotherapeutic drugs are used extensively for treating ESCC, including cisplatin (33, 34), docetaxel (33–35), nedaplatin (35), and fluorouracil (33–35), the prognosis of patients with ESCC is still very poor. Further development of some molecular drugs for ESCC is urgently required. In this study, it was the first time to identify ESCC mRNA, miRNA, and lncRNA modules by WGCNA at the same time. More importantly, the common mechanisms and molecular targets between ESCC and HNSCC were explored by bioinformatics analysis for the first time. We found six modules, including two mRNA modules (green and cyan modules), two miRNA modules (pink and purple modules), and two lncRNA modules (yellow and midnight blue modules), which were significantly related to the T stage of ESCC. We identified eight hub mRNAs, seven hub miRNAs, and 21 hub lncRNAs, and the lncRNA–miRNA–mRNA network was constructed. Moreover, the drugs targeting the prognostic factors were collected from DrugBank (https://www.drugbank.ca/). Most of the prognostic factors were not used to develop targeting drugs yet, and more studies need to be done. Recently, Pexidartinib, a molecular drug targeting CSF1R, was approved by the Food and Drug Administration in August 2019 as the first systemic therapy for adult patients with symptomatic tensynovial giant cell tumor (36). This achievement would provide the reference to our latter work. In the independent validation of prognostic biomarkers in independent dataset, all of the samples of GSE53625 were collected in China, while the samples of TCGA-ESCC were collected in America. The predictive capability of the biomarkers in cancer patients prognosis will be changed greatly in different races (37, 38). We speculated the predication performance of these biomarkers for ESCC are different in different races. In the future, we will further explore these biomarkers for ESCC *in vivo* and *in vitro* and compare the predictability of the prognostic biomarkers from different ethnic groups with more precision experimental methods.

Previous studies have revealed that esophageal cancer stage was more important in predicting outcome of synchronous ESCC/HNSCC patients (5, 39). The lncRNA–miRNA–mRNA network, which was based on the RNA modules related to T stage of ESCC, would help us understand the pathogenesis of ESCC-HNSCC. In this study, TBC1D2 and ATP6V0E1 were identified to be related to the T stage of ESCC, and they have a significantly better chance of becoming molecular factors for the prognosis prediction in ESCC-HNSCC. The expression levels of TBC1D2 and ATP6V0E1 were increased in both ESCC and HNSCC tissues, and they are closely related to the overall survival of ESCC and HNSCC, which means that TBC1D2 and ATP6V0E1 could be common therapeutic targets for both cancers.

Most interestingly, we found that the expression levels of SPI1, RNASE6 C1QB, C1QC, CSF1R, and C1QA were downregulated in ESCC, whereas they were upregulated in HNSCC. Some certain genes patriciate different molecular mechanisms in different tumor cells, so the expression levels of the genes would be very different (40, 41). We speculated that these genes participate in different pathogenesis in ESCC and HNSCC, thus making significantly different expression levels of these genes in different cancers. Functional data about how these genes participating in ESCC and HNSCC are not enough, and further studies are needed to explore the proposed mechanism for this interesting phenomenon.

As for the miR-515-5p and XIST related to the survival of ESCC, we conducted a literature review of them. miR-515-5p was initially described as a placenta-specific factor participating in fetal growth (42). Previous studies have identified its important role in breast cancer and non-small cell lung cancer (43, 44). miR-515-5p overexpression could inhibit cell migration in both lung and breast cancers, which demonstrated that miR-515-5p could be a target of some molecular drugs treating the metastatic cancer patients (44). In this study, it is the first time to discover that the expression level of miR-515-5p is negatively related to the overall survival of ESCC, and miR-515-5p might control cancer cell progression through RNASE6 regulation. As for the lncRNA XIST (X-inactive specific transcript), it is the master regulator of X inactivation and a product of the XIST gene (45). More and more research indicates that lncRNA XIST plays an important role in cell proliferation and differentiation, and it is dysregulated in many cancers (46, 47). A recent study demonstrated the abnormal expression of XIST could contribute to esophageal cancer via miR-494/CDK6 axis (48). We found that XIST might influence the prognosis of ESCC via miR-6756-5p/C1QA. Functional data about how XIST participates in cancer pathology are not enough, and further studies are needed.

The mRNAs in the hub modules were generally enriched in oxidation–reduction process and immune response. Cancer cell survival depends on various redox-related mechanisms, which are targets of currently developed therapies (49). Besides, disruption of redox homeostasis is a crucial factor in the development of drug resistance for ESCC, which is a major problem facing current cancer treatment (50). The genes in the hub modules would help us better understand the new resistance mechanism of the drugs for ESCC, such as paclitaxel, fluorouracil, and cisplatin. The immune system has an important role in the control of tumor outgrowth. Nowadays, immunotherapy is a novel treatment option that has shown encouraging efficacy in several types of cancer, also in ESCC, and early phase evaluation of immune checkpoint inhibitors has yielded promising results (51). The genes, playing an important role in immune response, might be new targets for cancer immunotherapy. The miRNAs and the lncRNAs in the hub modules were generally enriched in cell division and cell adhesion. A lot of cancer-promoting errors may occur during cell division, such as DNA mutations and epigenetic mistakes, chromosome aberrations occurring, and the wrong distribution of cell-fate determinants between the daughter cells (52, 53). The miRNAs and the lncRNAs in the hub modules might regulate the enzyme genes relating to cell division to control tumor cells division and growth in ESCC. Cell adhesion molecules are involved in a series of important physiological and pathological processes, such as cell signal transduction and activation, cell extension and movement, and tumor metastasis (54). The expression levels of important cell adhesion molecules are of great significance for disease diagnosis, guiding clinical therapy, and prognosis in ESCC (55). For example, the high expression of EGFR causes the abnormal differentiation of ESCC cells and the decrease in adhesion between cells, and the tumor is prone to lymphatic and distant metastasis (56, 57).

This work not only identify the prognostic factors of ESCC but also do further research for ESCC-HNSCC pathogenesis. WGCNA, GO/KEGG analysis, GSEA, and some databases (UALCAN, OncomiR, and OncoLnc) were used to fully explore the common mechanisms involving in ESCC and HNSCC. Useful clues were provided for clinical treatment of both diseases based on novel molecular advances, but there are still insufficient exist. First, nowadays, many studies tried to identify genes associated with progression and prognosis in patients with cancer using experimental methods. Lack of experiments (*in vivo* and *in vitro* validation) might be one limitation of our study. Second, the samples, suffering from ESCC and HNSCC, respectively, are not best one which is used to investigate mechanisms related to the prognosis of ESCC-HNSCC pathogenesis. We will further explore the ceRNA regulatory network and its role in the progression of ESCC-HNSCC using more in-depth bioinformatic analyses and experimental methods in the future.

In conclusion, the lncRNA–miRNA–mRNA network was conducted to explore the development of ESCC and common pathways between ESCC and HNSCC by WGCNA. We identified eight hub genes (TBC1D2, ATP6V0E1, SPI1, RNASE6, C1QB, C1QC, CSF1R, and C1QA), one hub miRNA (hsa-miR-515-5p), and one lncRNA (XIST), which might be prognostic biomarkers for ESCC. In the future, the pathogenic overlap of ESCC and HNSCC may help us to clarify the common molecular mechanisms between both diseases and may provide a potential treatment strategy for both diseases.

## Data Availability Statement

The datasets generated for this study can be found in the https://cancergenome.nih.gov/abouttcga/overview.

## Author Contributions

JHo and SL: conceived and designed the study. DY, XR, and JHu: performed the analysis procedures. DY, JHo, XR, CC, and YX: analyzed the results. WH and SL: contributed analysis tools. DY and JHo: contributed to the writing of the manuscript. All authors reviewed the manuscript.

### Conflict of Interest

The authors declare that the research was conducted in the absence of any commercial or financial relationships that could be construed as a potential conflict of interest.

## Supplementary Material

Figure S1 Determination of soft-thresholding power in the weighted gene co-expression network analysis (WGCNA). **(A)** Analysis of the scale-free fit index and the mean connectivity for various soft-thresholding powers for mRNA co-expression networks. **(B)** Analysis of the scale-free fit index and the mean connectivity for various soft-thresholding powers for miRNA co-expression networks. **(C)** Analysis of the scale-free fit index and the mean connectivity for various soft-thresholding powers for lncRNA co-expression networks.

Figure S2 Clustering dendrograms. **(A)** Clustering dendrograms of the mRNAs based on a dissimilarity measure (1-TOM). **(B)** Clustering dendrograms of miRNAs based on a dissimilarity measure (1-TOM). **(C)** Clustering dendrograms of lncRNAs based on a dissimilarity measure (1-TOM).

Figure S3 Protein-protein interaction networks for the genes in hub modules. **(A)** PPI network of 92 genes in cyan module. **(B)** PPI network of 279 genes in green module acquired from STRING 9.1.

Figure S4 Identification of hub mRNAs in ESCC based on |MM| in co-expression networks, degrees in PPI network, and survival analysis. **(A)** TBC1D2 and ATP6V0E1 were considered as real hub mRNAs in green module. **(B)** SPI1, RNASE6, C1QB, C1QC, CSF1R, and C1QA were considered as real hub mRNAs in cyan module.

Figure S5 Guilt of association for hub genes (SPI1, RNASE6, C1QB, C1QC, CSF1R, C1QA, TBC1D2, and ATP6V0E1).

Table S1 Gene expression microarray datasets related to ESCC.

Table S2 Node degree analysis for RNAs in ceRNA network.

Table S3 Gene set enriched in esophageal samples with TBC1D2 high expression.

Table S4 Gene set enriched in esophageal samples with ATP6V0E1 high expression.

Table S5 Gene set enriched in esophageal samples with SPI1 low expression.

Table S6 Gene set enriched in esophageal samples with RNASE6 low expression.

Table S7 Gene set enriched in esophageal samples with C1QB low expression.

Table S8 Gene set enriched in esophageal samples with C1QC low expression.

Table S9 Gene set enriched in esophageal samples with CSF1R low expression.

Table S10 Gene set enriched in esophageal samples with C1QA low expression.
