# Supplementary material for: Comprehensive Analysis of Competitive Endogenous RNAs Network, Being Associated With Esophageal Squamous Cell Carcinoma and Its Emerging Role in Head and Neck Squamous Cell Carcinoma
Source: Front Oncol. 2020 Jan 21;9:1474. doi: 10.3389/fonc.2019.01474 (PMC6985543; doi:10.3389/fonc.2019.01474)
Supplement: Figure S1 — Determination of soft-thresholding power in the weighted gene co-expression network analysis (WGCNA). (A) Analysis of the scale-free fit index and the mean connectivity for various soft-thresholding powers for mRNA co-expression networks. (B) Analysis of the scale-free fit index and the mean connectivity for various soft-thresholding powers for miRNA co-expression networks. (C) Analysis of the scale-free fit index and the mean connectivity for various soft-thresholding powers for lncRNA co-expression networks. [file Data_Sheet_1.ZIP › Supplementary materials/Table S1.docx]

**Table1:** **Gene expression microarray datasets related to ESCC.**

| Accession number of the dataset | Platform | Submission date | Organism | Disease type | |
| --- | --- | --- | --- | --- | --- |
|  |  |  |  | Control | ESCC |
| GSE20347 | GPL571 | Feb 16, 2010 | Homo sapiens | 17 | 17 |
| GSE38129 | GPL571 | May 22, 2012 | Homo sapiens | 30 | 30 |

Note. ESCC, esophageal squamous cell carcinoma.
