# Supplementary material for: Comprehensive Analysis of Competitive Endogenous RNAs Network, Being Associated With Esophageal Squamous Cell Carcinoma and Its Emerging Role in Head and Neck Squamous Cell Carcinoma
Source: Front Oncol. 2020 Jan 21;9:1474. doi: 10.3389/fonc.2019.01474 (PMC6985543; doi:10.3389/fonc.2019.01474)
Supplement: Figure S1 — Determination of soft-thresholding power in the weighted gene co-expression network analysis (WGCNA). (A) Analysis of the scale-free fit index and the mean connectivity for various soft-thresholding powers for mRNA co-expression networks. (B) Analysis of the scale-free fit index and the mean connectivity for various soft-thresholding powers for miRNA co-expression networks. (C) Analysis of the scale-free fit index and the mean connectivity for various soft-thresholding powers for lncRNA co-expression networks. [file Data_Sheet_1.ZIP › Supplementary materials/Table S10.docx]

**Table S10**: **Gene set enriched in esophageal samples with C1QA low expression.**

| C1QA | SIZE | ES | NES | NOM  p-value | FDR  q-value |
| --- | --- | --- | --- | --- | --- |
| Positive regulation of leukocyte proliferation | 134 | 0.670838 | 2.481786 | 0 | 0 |
| Regulation of leukocyte proliferation | 201 | 0.634276 | 2.43378 | 0 | 0 |
| Lymphocyte mediated immunity | 116 | 0.630356 | 2.431445 | 0 | 0 |
| Regulation of adaptive immune response | 123 | 0.647766 | 2.400097 | 0 | 0.000154 |
| Adaptive immune response based on somatic recombination of immune receptors built from immunoglobulin superfamily domains | 123 | 0.635385 | 2.389819 | 0 | 0.000123 |
| Response to interferon gamma | 139 | 0.690215 | 2.375909 | 0 | 7.69E-05 |
| Regulation of t cell proliferation | 143 | 0.633764 | 2.364431 | 0 | 6.84E-05 |
| Adaptive immune response | 251 | 0.659256 | 2.344065 | 0 | 5.59E-05 |
| Cellular response to interferon gamma | 117 | 0.694636 | 2.338433 | 0 | 5.35E-05 |
| Regulation of leukocyte mediated immunity | 156 | 0.618727 | 2.305444 | 0 | 0.000129 |
| Regulation of lymphocyte mediated immunity | 114 | 0.632583 | 2.29466 | 0 | 0.000202 |
| Leukocyte chemotaxis | 114 | 0.627029 | 2.247785 | 0 | 0.000476 |

Note. ES, enrichment score; NES, normalized enrichment score; NOM p-value, nominal p value; FDR, false discovery rate q value.
