# Supplementary material for: Comprehensive Analysis of Competitive Endogenous RNAs Network, Being Associated With Esophageal Squamous Cell Carcinoma and Its Emerging Role in Head and Neck Squamous Cell Carcinoma
Source: Front Oncol. 2020 Jan 21;9:1474. doi: 10.3389/fonc.2019.01474 (PMC6985543; doi:10.3389/fonc.2019.01474)
Supplement: Figure S1 — Determination of soft-thresholding power in the weighted gene co-expression network analysis (WGCNA). (A) Analysis of the scale-free fit index and the mean connectivity for various soft-thresholding powers for mRNA co-expression networks. (B) Analysis of the scale-free fit index and the mean connectivity for various soft-thresholding powers for miRNA co-expression networks. (C) Analysis of the scale-free fit index and the mean connectivity for various soft-thresholding powers for lncRNA co-expression networks. [file Data_Sheet_1.ZIP › Supplementary materials/Table S2.docx]

Table S2. Node degree analysis for RNAs in ceRNA network.

|  | Name | Degree | Topological coefficient |
| --- | --- | --- | --- |
| miRNA | hsa-miR-519e-5p | 10 | 0.25 |
| miRNA | hsa-miR-515-5p | 9 | 0.259259 |
| miRNA | hsa-miR-6756-5p | 8 | 0.138889 |
| mRNA | C1QA | 7 | 0.261905 |
| mRNA | SPI1 | 5 | 0.48 |
| mRNA | RNASE6 | 5 | 0.355556 |
| miRNA | hsa-miR-6769b-5p | 5 | 0.228571 |
| miRNA | hsa-miR-650 | 5 | 0.2 |
| mRNA | C1QB | 4 | 0.714286 |
| mRNA | C1QC | 4 | 0.714286 |
| mRNA | CSF1R | 4 | 0.714286 |
| miRNA | hsa-miR-519d-5p | 4 | 0.3 |
| lncRNA | XIST | 3 | 0.5 |
| lncRNA | RP11-440L14.1 | 3 | 0.6 |
| miRNA | hsa-miR-4707-3p | 2 | 0.5 |
| lncRNA | ATP6V0E1 | 2 | 0.5 |
| lncRNA | CTD-2023N9.1 | 2 | 0.944444 |
| lncRNA | RP5-1184F4.5 | 2 | 0.944444 |
| lncRNA | RP11-332H14.2 | 2 | 0.944444 |
| lncRNA | ETV5-AS1 | 2 | 0.944444 |
| lncRNA | RP11-327F22.6 | 2 | 0.545455 |
| lncRNA | AC141928.1 | 2 | 0.944444 |
| lncRNA | AC016735.1 | 2 | 0.944444 |
| lncRNA | AC226118.1 | 2 | 0.55 |
| lncRNA | C1orf213 | 2 | 0.5 |
| lncRNA | CTD-3018O17.3 | 2 | 0.5 |
| mRNA | TBC1D2 | 1 | 0 |
| lncRNA | RP5-1054A22.4 | 1 | 0 |
| lncRNA | RP5-1029K10.2 | 1 | 0 |
| lncRNA | PSMG3-AS1 | 1 | 0 |
| lncRNA | RP5-1125A11.7 | 1 | 0 |
| lncRNA | LINC01355 | 1 | 0 |
| lncRNA | RP11-2H3.6 | 1 | 0 |
| lncRNA | RP11-275I4.2 | 1 | 0 |
| lncRNA | RP3-470B24.5 | 1 | 0 |
| lncRNA | RP11-504P24.8 | 1 | 0 |

Note. miRNA, microRNA. lncRNA, long non-coding RNA.
