# Supplementary material for: Comprehensive Analysis of Competitive Endogenous RNAs Network, Being Associated With Esophageal Squamous Cell Carcinoma and Its Emerging Role in Head and Neck Squamous Cell Carcinoma
Source: Front Oncol. 2020 Jan 21;9:1474. doi: 10.3389/fonc.2019.01474 (PMC6985543; doi:10.3389/fonc.2019.01474)
Supplement: Figure S1 — Determination of soft-thresholding power in the weighted gene co-expression network analysis (WGCNA). (A) Analysis of the scale-free fit index and the mean connectivity for various soft-thresholding powers for mRNA co-expression networks. (B) Analysis of the scale-free fit index and the mean connectivity for various soft-thresholding powers for miRNA co-expression networks. (C) Analysis of the scale-free fit index and the mean connectivity for various soft-thresholding powers for lncRNA co-expression networks. [file Data_Sheet_1.ZIP › Supplementary materials/Table S3.docx]

**Table S3**: **Gene set enriched in esophageal samples with TBC1D2 high expression.**

| TBC1D2 | SIZE | ES | NES | NOM  p-value | FDR  q-value |
| --- | --- | --- | --- | --- | --- |
| Translational initiation | 143 | 0.671402 | 1.801917 | 0.036965 | 0.135406 |
| Multi organism metabolic process | 137 | 0.632084 | 1.756309 | 0.047151 | 0.219462 |
| Establishment of protein localization to endoplasmic reticulum | 104 | 0.704928 | 1.695145 | 0.053254 | 0.153073 |
| Protein localization to endoplasmic reticulum | 123 | 0.64268 | 1.684847 | 0.072835 | 0.149368 |

Note. ES, enrichment score; NES, normalized enrichment score; NOM p-value, nominal p value; FDR, false discovery rate q value.
