# Supplementary material for: Comprehensive Analysis of Competitive Endogenous RNAs Network, Being Associated With Esophageal Squamous Cell Carcinoma and Its Emerging Role in Head and Neck Squamous Cell Carcinoma
Source: Front Oncol. 2020 Jan 21;9:1474. doi: 10.3389/fonc.2019.01474 (PMC6985543; doi:10.3389/fonc.2019.01474)
Supplement: Figure S1 — Determination of soft-thresholding power in the weighted gene co-expression network analysis (WGCNA). (A) Analysis of the scale-free fit index and the mean connectivity for various soft-thresholding powers for mRNA co-expression networks. (B) Analysis of the scale-free fit index and the mean connectivity for various soft-thresholding powers for miRNA co-expression networks. (C) Analysis of the scale-free fit index and the mean connectivity for various soft-thresholding powers for lncRNA co-expression networks. [file Data_Sheet_1.ZIP › Supplementary materials/Table S4.docx]

**Table S4**: **Gene set enriched in esophageal samples with ATP6V0E1 high expression.**

| ATP6V0E1 | SIZE | ES | NES | NOM  p-value | FDR  q-value |
| --- | --- | --- | --- | --- | --- |
| Amide biosynthetic process | 490 | 0.610313 | 2.197444 | 0.001919 | 0.014573 |
| Nucleoside triphosphate metabolic process | 217 | 0.609535 | 2.161811 | 0 | 0.011208 |
| Cellular respiration | 138 | 0.688835 | 2.158912 | 0 | 0.010488 |
| Protein localization to endoplasmic reticulum | 123 | 0.791987 | 2.092513 | 0.001992 | 0.012076 |
| Protein targeting to membrane | 156 | 0.680063 | 2.086989 | 0.004049 | 0.0128 |
| Translational initiation | 143 | 0.76619 | 2.041157 | 0.005871 | 0.01862 |
| Nuclear transcribed mRNA catabolic process nonsense mediated decay | 118 | 0.773195 | 2.032803 | 0.003953 | 0.020054 |
| Translational elongation | 110 | 0.697353 | 2.008967 | 0.001961 | 0.023057 |
| Multi organism metabolic process | 137 | 0.715647 | 1.979914 | 0.00994 | 0.027021 |
| Establishment of protein localization to endoplasmic reticulum | 104 | 0.817185 | 1.944407 | 0.002028 | 0.034245 |
| Mitochondrial translation | 105 | 0.699104 | 1.935568 | 0.005882 | 0.036071 |
| Cellular protein complex disassembly | 121 | 0.603654 | 1.926389 | 0.007797 | 0.036999 |
| rRNA metabolic process | 250 | 0.624066 | 1.890735 | 0.00789 | 0.04546 |

Note. ES, enrichment score; NES, normalized enrichment score; NOM p-value, nominal p value; FDR, false discovery rate q value.
