# Supplementary material for: Comprehensive Analysis of Competitive Endogenous RNAs Network, Being Associated With Esophageal Squamous Cell Carcinoma and Its Emerging Role in Head and Neck Squamous Cell Carcinoma
Source: Front Oncol. 2020 Jan 21;9:1474. doi: 10.3389/fonc.2019.01474 (PMC6985543; doi:10.3389/fonc.2019.01474)
Supplement: Figure S1 — Determination of soft-thresholding power in the weighted gene co-expression network analysis (WGCNA). (A) Analysis of the scale-free fit index and the mean connectivity for various soft-thresholding powers for mRNA co-expression networks. (B) Analysis of the scale-free fit index and the mean connectivity for various soft-thresholding powers for miRNA co-expression networks. (C) Analysis of the scale-free fit index and the mean connectivity for various soft-thresholding powers for lncRNA co-expression networks. [file Data_Sheet_1.ZIP › Supplementary materials/Table S5.docx]

**Table S5**: **Gene set enriched in esophageal samples with SPI1 low expression.**

| SPI1 | SIZE | ES | NES | NOM  p-value | FDR  q-value |
| --- | --- | --- | --- | --- | --- |
| Positive regulation of leukocyte proliferation | 134 | 0.676078 | 2.497362 | 0 | 0 |
| Adaptive immune response based on somatic recombination of immune receptors built from immunoglobulin superfamily domains | 123 | 0.667604 | 2.473124 | 0 | 0 |
| Regulation of adaptive immune response | 123 | 0.662663 | 2.443945 | 0 | 0 |
| Lymphocyte mediated immunity | 116 | 0.634539 | 2.430237 | 0 | 0 |
| Regulation of leukocyte proliferation | 201 | 0.632369 | 2.429504 | 0 | 0 |
| Positive regulation of cell activation | 283 | 0.601862 | 2.410489 | 0 | 0 |
| Positive regulation of cell adhesion | 234 | 0.602482 | 2.403957 | 0 | 0 |
| Adaptive immune response | 251 | 0.665481 | 2.39661 | 0 | 0 |
| Response to interferon gamma | 139 | 0.693161 | 2.376415 | 0 | 0.000105 |
| Antigen receptor mediated signaling pathway | 169 | 0.612689 | 2.367829 | 0 | 0.000161 |
| Regulation of lymphocyte mediated immunity | 114 | 0.648142 | 2.364877 | 0 | 0.000146 |
| Regulation of T cell proliferation | 143 | 0.632727 | 2.361163 | 0 | 0.000128 |
| Regulation of leukocyte mediated immunity | 156 | 0.623538 | 2.359431 | 0 | 0.000115 |
| Cellular response to interferon gamma | 117 | 0.708065 | 2.331769 | 0 | 0.000203 |
| Regulation of B cell activation | 102 | 0.612616 | 2.278691 | 0 | 0.000289 |

Note. ES, enrichment score; NES, normalized enrichment score; NOM p-value, nominal p value; FDR, false discovery rate q value.
