# Supplementary material for: Comprehensive Analysis of Competitive Endogenous RNAs Network, Being Associated With Esophageal Squamous Cell Carcinoma and Its Emerging Role in Head and Neck Squamous Cell Carcinoma
Source: Front Oncol. 2020 Jan 21;9:1474. doi: 10.3389/fonc.2019.01474 (PMC6985543; doi:10.3389/fonc.2019.01474)
Supplement: Figure S1 — Determination of soft-thresholding power in the weighted gene co-expression network analysis (WGCNA). (A) Analysis of the scale-free fit index and the mean connectivity for various soft-thresholding powers for mRNA co-expression networks. (B) Analysis of the scale-free fit index and the mean connectivity for various soft-thresholding powers for miRNA co-expression networks. (C) Analysis of the scale-free fit index and the mean connectivity for various soft-thresholding powers for lncRNA co-expression networks. [file Data_Sheet_1.ZIP › Supplementary materials/Table S6.docx]

**Table S6**: **Gene set enriched in esophageal samples with RNASE6 low expression.**

| RNASE6 | SIZE | ES | NES | NOM  p-value | FDR  q-value |
| --- | --- | --- | --- | --- | --- |
| Adaptive immune response based on somatic recombination of immune receptors built from immunoglobulin superfamily domains | 123 | 0.688493 | 2.59488 | 0 | 0 |
| Lymphocyte mediated immunity | 116 | 0.670038 | 2.568806 | 0 | 0 |
| Regulation of adaptive immune response | 123 | 0.691145 | 2.552649 | 0 | 0 |
| Adaptive immune response | 251 | 0.703491 | 2.518697 | 0 | 0.000237 |
| Positive regulation of cell activation | 283 | 0.62809 | 2.51711 | 0 | 0.000178 |
| Regulation of lymphocyte mediated immunity | 114 | 0.684654 | 2.515915 | 0 | 0.000158 |
| Antigen receptor mediated signaling pathway | 169 | 0.642273 | 2.507012 | 0 | 0.000142 |
| Positive regulation of leukocyte proliferation | 134 | 0.682426 | 2.501617 | 0 | 0.000129 |
| Leukocyte activation | 409 | 0.602125 | 2.501057 | 0 | 0.000109 |
| Positive regulation of cell adhesion | 234 | 0.619488 | 2.499066 | 0 | 0.000102 |
| Regulation of homotypic cell adhesion | 294 | 0.60646 | 2.496276 | 0 | 8.89E-05 |
| Leukocyte mediated immunity | 157 | 0.628305 | 2.475228 | 0 | 7.12E-05 |
| Lymphocyte activation | 340 | 0.603906 | 2.473475 | 0 | 6.47E-05 |
| Regulation of T cell proliferation | 143 | 0.648705 | 2.470053 | 0 | 6.19E-05 |
| Regulation of leukocyte proliferation | 201 | 0.638913 | 2.4604 | 0 | 5.93E-05 |
| Leukocyte cell adhesion | 254 | 0.608598 | 2.421457 | 0 | 9.38E-05 |
| Regulation of leukocyte mediated immunity | 156 | 0.644562 | 2.41083 | 0 | 0.000129 |
| T cell receptor signaling pathway | 139 | 0.611751 | 2.357863 | 0 | 0.000287 |
| Cellular response to interferon gamma | 117 | 0.686858 | 2.343152 | 0 | 0.000313 |
| T cell differentiation | 123 | 0.617313 | 2.335488 | 0 | 0.000362 |
| Response to interferon gamma | 139 | 0.670431 | 2.327201 | 0 | 0.000405 |
| Negative regulation of immune response | 117 | 0.606964 | 2.314732 | 0 | 0.000471 |
| Regulation of B cell activation | 102 | 0.629246 | 2.302906 | 0 | 0.000509 |
| Leukocyte chemotaxis | 114 | 0.620179 | 2.246125 | 0 | 0.000853 |
| Positive regulation of chemotaxis | 119 | 0.611747 | 2.21954 | 0 | 0.001019 |

Note. ES, enrichment score; NES, normalized enrichment score; NOM p-value, nominal p value; FDR, false discovery rate q value.
