# Supplementary material for: Comprehensive Analysis of Competitive Endogenous RNAs Network, Being Associated With Esophageal Squamous Cell Carcinoma and Its Emerging Role in Head and Neck Squamous Cell Carcinoma
Source: Front Oncol. 2020 Jan 21;9:1474. doi: 10.3389/fonc.2019.01474 (PMC6985543; doi:10.3389/fonc.2019.01474)
Supplement: Figure S1 — Determination of soft-thresholding power in the weighted gene co-expression network analysis (WGCNA). (A) Analysis of the scale-free fit index and the mean connectivity for various soft-thresholding powers for mRNA co-expression networks. (B) Analysis of the scale-free fit index and the mean connectivity for various soft-thresholding powers for miRNA co-expression networks. (C) Analysis of the scale-free fit index and the mean connectivity for various soft-thresholding powers for lncRNA co-expression networks. [file Data_Sheet_1.ZIP › Supplementary materials/Table S7.docx]

**Table S7**: **Gene set enriched in esophageal samples with C1QB low expression.**

| C1QB | SIZE | ES | NES | NOM  p-value | FDR  q-value |
| --- | --- | --- | --- | --- | --- |
| Response to interferon gamma | 139 | 0.704469 | 2.467176 | 0 | 0 |
| Regulation of leukocyte proliferation | 201 | 0.633556 | 2.445192 | 0 | 0 |
| Regulation of adaptive immune response | 123 | 0.655612 | 2.441899 | 0 | 0 |
| Positive regulation of leukocyte proliferation | 134 | 0.66613 | 2.440589 | 0 | 0 |
| Cellular response to interferon gamma | 117 | 0.710047 | 2.399042 | 0 | 0.000233 |
| Lymphocyte mediated immunity | 116 | 0.6289 | 2.388267 | 0 | 0.000186 |
| Regulation of leukocyte mediated immunity | 156 | 0.62726 | 2.387118 | 0 | 0.000174 |
| Adaptive immune response | 251 | 0.658029 | 2.384378 | 0 | 0.000164 |
| Regulation of T cell proliferation | 143 | 0.634648 | 2.381698 | 0 | 0.000155 |
| Adaptive immune response based on somatic Recombination of immune receptors built from immunoglobulin superfamily domains | 123 | 0.627698 | 2.378108 | 0 | 0.000147 |
| Regulation of lymphocyte mediated immunity | 114 | 0.649607 | 2.349034 | 0 | 0.000243 |
| Antigen receptor mediated signaling pathway | 169 | 0.606698 | 2.322257 | 0 | 0.000394 |
| Antigen processing and presentation of peptide antigen | 170 | 0.601305 | 2.285182 | 0 | 0.000434 |
| Leukocyte chemotaxis | 114 | 0.619376 | 2.236661 | 0 | 0.000898 |

Note. ES, enrichment score; NES, normalized enrichment score; NOM p-value, nominal p value; FDR, false discovery rate q value.
