# Supplementary material for: Comprehensive Analysis of Competitive Endogenous RNAs Network, Being Associated With Esophageal Squamous Cell Carcinoma and Its Emerging Role in Head and Neck Squamous Cell Carcinoma
Source: Front Oncol. 2020 Jan 21;9:1474. doi: 10.3389/fonc.2019.01474 (PMC6985543; doi:10.3389/fonc.2019.01474)
Supplement: Figure S1 — Determination of soft-thresholding power in the weighted gene co-expression network analysis (WGCNA). (A) Analysis of the scale-free fit index and the mean connectivity for various soft-thresholding powers for mRNA co-expression networks. (B) Analysis of the scale-free fit index and the mean connectivity for various soft-thresholding powers for miRNA co-expression networks. (C) Analysis of the scale-free fit index and the mean connectivity for various soft-thresholding powers for lncRNA co-expression networks. [file Data_Sheet_1.ZIP › Supplementary materials/Table S8.docx]

**Table S8**: **Gene set enriched in esophageal samples with C1QC low expression.**

| C1QC | SIZE | ES | NES | NOM p-value | FDR  q-value |
| --- | --- | --- | --- | --- | --- |
| Lymphocyte mediated immunity | 116 | 0.656506 | 2.498159 | 0 | 0 |
| Regulation of leukocyte proliferation | 201 | 0.642356 | 2.441781 | 0 | 0 |
| Leukocyte mediated immunity | 157 | 0.627271 | 2.436482 | 0 | 0 |
| Positive regulation of leukocyte proliferation | 134 | 0.672886 | 2.429126 | 0 | 0 |
| Adaptive immune response based on somatic recombination of immune receptors built from immunoglobulin superfamily domains | 123 | 0.657668 | 2.404149 | 0 | 0.000105 |
| Positive regulation of cell activation | 283 | 0.616842 | 2.402054 | 0 | 9.77E-05 |
| Antigen receptor mediated signaling pathway | 169 | 0.631195 | 2.398581 | 0 | 8.55E-05 |
| Regulation of adaptive immune response | 123 | 0.658409 | 2.386084 | 0 | 7.6E-05 |
| Adaptive immune response | 251 | 0.680931 | 2.376321 | 0 | 7.2E-05 |
| Positive regulation of cell adhesion | 234 | 0.600073 | 2.359606 | 0 | 5.7E-05 |
| Regulation of leukocyte mediated immunity | 156 | 0.634703 | 2.35339 | 0 | 5.26E-05 |
| Response to interferon gamma | 139 | 0.704271 | 2.351472 | 0 | 5.07E-05 |
| Cellular response to interferon gamma | 117 | 0.70637 | 2.329435 | 0 | 0.000109 |
| Regulation of B cell activation | 102 | 0.640894 | 2.304483 | 0 | 0.000207 |
| Regulation of T cell proliferation | 143 | 0.630737 | 2.28948 | 0 | 0.000259 |
| Regulation of lymphocyte mediated immunity | 114 | 0.641647 | 2.287198 | 0 | 0.000254 |
| Leukocyte chemotaxis | 114 | 0.603337 | 2.101775 | 0 | 0.00364 |

Note. ES, enrichment score; NES, normalized enrichment score; NOM p-value, nominal p value; FDR, false discovery rate q value.
