# Supplementary material for: Comprehensive Analysis of Competitive Endogenous RNAs Network, Being Associated With Esophageal Squamous Cell Carcinoma and Its Emerging Role in Head and Neck Squamous Cell Carcinoma
Source: Front Oncol. 2020 Jan 21;9:1474. doi: 10.3389/fonc.2019.01474 (PMC6985543; doi:10.3389/fonc.2019.01474)
Supplement: Figure S1 — Determination of soft-thresholding power in the weighted gene co-expression network analysis (WGCNA). (A) Analysis of the scale-free fit index and the mean connectivity for various soft-thresholding powers for mRNA co-expression networks. (B) Analysis of the scale-free fit index and the mean connectivity for various soft-thresholding powers for miRNA co-expression networks. (C) Analysis of the scale-free fit index and the mean connectivity for various soft-thresholding powers for lncRNA co-expression networks. [file Data_Sheet_1.ZIP › Supplementary materials/Table S9.docx]

**Table S9**: **Gene set enriched in esophageal samples with CSF1R low expression.**

| CSF1R | SIZE | ES | NES | NOM p-value | FDR  q-value |
| --- | --- | --- | --- | --- | --- |
| Regulation of homotypic cell adhesion | 294 | 0.605861 | 2.516969 | 0 | 0 |
| Positive regulation of cell adhesion | 234 | 0.611032 | 2.514198 | 0 | 0 |
| Positive regulation of cell activation | 283 | 0.619981 | 2.507999 | 0 | 0 |
| Regulation of leukocyte proliferation | 201 | 0.631602 | 2.487293 | 0 | 0 |
| Regulation of T cell proliferation | 143 | 0.646675 | 2.477286 | 0 | 0 |
| Negative regulation of cell activation | 153 | 0.602105 | 2.435374 | 0 | 0 |
| Adaptive immune response based on somatic recombination of immune receptors built from immunoglobulin superfamily domains | 123 | 0.639481 | 2.425953 | 0 | 0 |
| Positive regulation of leukocyte proliferation | 134 | 0.651671 | 2.416074 | 0 | 0 |
| Lymphocyte mediated immunity | 116 | 0.61283 | 2.388671 | 0 | 0 |
| Adaptive immune response | 251 | 0.657569 | 2.370327 | 0 | 4.99E-05 |
| Regulation of B cell activation | 102 | 0.624353 | 2.321907 | 0 | 0.000102 |
| Regulation of leukocyte mediated immunity | 156 | 0.614688 | 2.313565 | 0 | 9.95E-05 |
| Regulation of lymphocyte mediated immunity | 114 | 0.6276 | 2.293193 | 0 | 0.000147 |
| Cellular response to interferon gamma | 117 | 0.672722 | 2.276658 | 0 | 0.000223 |
| Response to interferon gamma | 139 | 0.656912 | 2.275178 | 0 | 0.000236 |
| Regulation of adaptive immune response | 123 | 0.615039 | 2.25138 | 0 | 0.000358 |

Note. ES, enrichment score; NES, normalized enrichment score; NOM p-value, nominal p value; FDR, false discovery rate q value.
